# Meconium Fatty Acid Ethyl Esters as Biomarkers of Late Gestational Ethanol Exposure and Indicator of Ethanol-Induced Multi-Organ Injury in Fetal Sheep

**DOI:** 10.1371/journal.pone.0059168

**Published:** 2013-03-22

**Authors:** Irene Zelner, Kelly Kenna, James F. Brien, Alan Bocking, Richard Harding, David Walker, Gideon Koren

**Affiliations:** 1 Division of Clinical Pharmacology and Toxicology, The Hospital for Sick Children, Toronto, Ontario, Canada; 2 Department of Pharmacology and Toxicology, University of Toronto, Toronto, Ontario; 3 Department of Anatomy and Developmental Biology, Monash University, Clayton, Victoria, Australia; 4 Department of Biomedical and Molecular Sciences, Queen’s University, Kingston, Ontario, Canada; 5 Department of Obstetrics and Gynaecology, University of Toronto, Toronto, Ontario, Canada; 6 Ritchie Centre for Infant Health, Monash Institute of Medical Research, Monash University, Clayton, Victoria, Australia; Emory University School of Medicine, United States of America

## Abstract

**Background:**

Meconium fatty acid ethyl esters (FAEE) constitute a biomarker of heavy fetal ethanol exposure. Our objective was to measure meconium FAEE in fetal sheep following daily, relatively moderate-dose ethanol exposure in late gestation, and to evaluate their utility in identifying fetal organ-system injury.

**Methods:**

Pregnant ewes received ethanol (0.75 g/kg; *n* = 14) or saline (*n* = 8) via 1-h IV infusion daily during the third trimester equivalent, while additional pregnant sheep served as untreated controls (*n* = 6). The daily ethanol regimen produced similar maximal maternal and fetal plasma ethanol concentrations of 0.11–0.12 g/dL. Ewes and fetuses were euthanized shortly before term, and meconium was collected and analyzed for FAEE (ethyl palmitate, stearate, linoleate, and oleate).

**Results:**

Meconium total FAEE concentration was significantly higher in ethanol-exposed fetuses compared with controls, and a positive cut-off of 0.0285 nmol total FAEE/g meconium had 93.3% sensitivity and specificity for detecting fetal ethanol exposure. When the studied animals (ethanol-exposed and controls) were classified according to meconium FAEE concentration, FAEE-positive and FAEE-negative groups frequently differed with respect to previously examined pathological endpoints, including nephron endowment, lung collagen deposition, cardiomyocyte maturation, and tropoelastin gene expression in cerebral vessels. Furthermore, in all studied animals as a group (ethanol-exposed and controls combined), meconium FAEE concentration was correlated with many of these pathological endpoints in fetal organs.

**Conclusions:**

We conclude that, in fetal sheep, meconium FAEE could serve as a biomarker of daily ethanol exposure in late gestation and could identify fetuses with subtle ethanol-induced toxic effects in various organs. This study illustrates the potential for using meconium FAEE to identify neonates at risk for dysfunction of major organs following *in-utero* ethanol exposure that does not result in overt physical signs of ethanol teratogenicity.

## Introduction

Alcohol (ethanol) is a well established human teratogen that can result in a range of physical defects, and cognitive and behavioral deficits, known collectively as fetal alcohol spectrum disorders (FASD) [1]. The effects of prenatal alcohol exposure can range in severity from mild to debilitating, with the Fetal Alcohol Syndrome (the most severe end of the spectrum) having the principal features of growth restriction, characteristic craniofacial dysmorphology, and central nervous system dysfunction, including intellectual, neurological and behavioural deficits [2]–[4]. Dosage regimen, gestational timing of alcohol exposure, as well as maternal and fetal characteristics, contribute to the multiplicity of the FASD phenotype that presents in postnatal life. Prenatal alcohol exposure is considered a leading known cause of developmental disability, with FASD affecting as many as 1–5% of children in North America and Western European countries [5], [6].

A major challenge in diagnosing milder forms of FASD is the absence of clear external markers of ethanol teratogenicity such as the craniofacial characteristics, which are present only in the most severe cases. In the absence of such overt dysmorphology, it is necessary to confirm prenatal alcohol exposure to make a diagnosis [7]. Because of the poor sensitivity and reliability of maternal self-reporting of alcohol consumption [8], this is often problematic and can lead to misdiagnosis and/or late recognition of FASD. Since early diagnosis and intervention are associated with improved outcomes and decreased secondary disabilities in individuals with a FASD [9], the development of a reliable biomarker of fetal ethanol exposure is therefore of great clinical importance as it can enable the early recognition of at-risk individuals, timely diagnosis, and implementation of interventions.

Fatty acid ethyl esters (FAEE) are produced by non-oxidative ethanol biotransformation involving the conjugation of ethanol to endogenous free fatty acids or fatty-acyl-CoA [10]. Unlike ethanol and its proximate metabolite, acetaldehyde, FAEE have a prolonged half-life and accumulate in body tissues and fluids. One matrix in which FAEE accumulate and can be measured is fetal meconium, which begins to form in the fetal intestine with the emergence of fetal swallowing of amniotic fluid around 15 weeks of gestation [11]. In humans, FAEE in neonatal meconium have been validated as sensitive and specific biomarkers of heavy maternal drinking in the latter half of pregnancy [12]–[14], and a recent Canadian FASD steering committee has concluded that analysis of FAEE in meconium may be a useful screening tool for the identification of newborns at risk for FASD [15]. However, the sensitivity and specificity of meconium FAEE for detecting lower levels of fetal ethanol exposure, and their ability to identify individuals with subtle manifestations of organ or system injury are not clear.

Recently, a comprehensive study was conducted on the effects of repeated maternal administration of relatively moderate-dose ethanol in pregnant sheep during the third-trimester-equivalent on several fetal organs. In this study, pregnant ewes received one-hour daily ethanol infusion during the third-trimester-equivalent that produced maximal maternal and fetal plasma ethanol concentrations (PEC) of 0.11–0.12 g/dL, which approximated blood ethanol concentration measured in non-pregnant women drinking socially (equivalent to a 55–70 kg woman consuming 3–4 standard drinks over one hour) [16]. This maternal ethanol exposure regimen did not result in overt fetal dysmorphology, but ethanol-induced changes were found in several fetal organs examined, including the heart [17], kidney [18], lung [19], brain and placenta [20]. As meconium was collected from these fetuses, our objective was to determine whether meconium FAEE concentration is a biomarker of repeated fetal exposure to this ethanol regimen in the third-trimester-equivalent of ovine pregnancy. Furthermore, we also assessed the relationship between meconium FAEE concentration (biomarker of exposure) and fetal organ injury (effect) in the studied animals. Our results suggest that meconium FAEE concentrations could serve as a biomarker of daily ethanol exposure in late gestation, and could be used to identify fetuses with subtle ethanol-induced multi-organ pathology, thereby supporting the potential utility of meconium testing as a screening tool for the identification of newborns at risk for FASD.

## Materials and Methods

### Ethics Statement

All animal experiments were approved by the Animal Ethics Committee of Monash University where the animal experimentation was performed (permit number: PHYS/2005/31) in accordance with the National Health and Medical Research Council of Australia guidelines. All surgery was performed under anesthesia as described below, and all efforts were made to minimize suffering.

### Research Plan

Experimental animal preparation, ethanol dosing, and study design have been described previously in detail [17]–[20]. The treatment groups are presented in **Table 1**. Briefly, at 90–91 days gestational age (DGA; full term ∼147 DGA), 22 pregnant Merino X Border-Leicester ewes were anesthetized with intravenous thiopental sodium (1 g) and maintained by inhalation of 1–2% halothane in O_2_-N_2_O (50∶50 vol/vol). Catheters were aseptically inserted into a maternal carotid artery for blood sampling and into a jugular vein for ethanol or saline infusion. Six additional sheep served as untouched controls. From 95–124 DGA, 14 randomly chosen surgically-instrumented pregnant ewes received daily, 1-h intravenous infusion of 0.75 g ethanol/kg maternal body weight of 40% (v/v) ethanol-saline solution, and 8 pregnant ewes received 1-h intravenous infusion of an equivalent volume of saline. Pregnant ewes had free access to food and water, and animals in the saline cohort were provided with about 120 g (∼250 calories) of additional food to compensate for calories derived from ethanol metabolism. At 126 DGA, pregnant ewes (n = 8 ethanol and n = 8 saline group) were anesthetized, and catheters were inserted aseptically into the fetal brachial artery for fetal blood collection. During fetal surgery twins were observed in one ethanol and one control animal, and only one of the twins was catheterized. After recovery from surgery, daily maternal ethanol infusion resumed on 128 DGA and continued until 133 DGA. Paired fetal and maternal arterial blood samples were collected from 131–134 DGA and it was determined that this ethanol regimen produced maximal maternal and fetal PEC of 0.12±0.01 g/dL and 0.11±0.01 g/dL (mean +/− SEM), respectively, at the end of the 1-h ethanol infusion [17]–[20].

**Table 1:** Animal treatment groups.

Group	MaternalSurgery^a^	Treatment^b^	FetalSurgery^c^	Ewe (*n*)	Fetuses (*n*)
1	Yes	Ethanol	Yes	8	9
2	Yes	Saline	Yes	8	9
3	Yes	Ethanol	No	6	6
4	No	None	No	6	6

a Surgical implantation of catheters into a maternal carotid artery for blood sampling and into a jugular vein for ethanol or saline infusion at 90–91 days gestational age (DGA).

b Daily 1-h infusion of ethanol (0.75 g/kg) or saline from 95–133 DGA.

c Surgical implantation of catheter into fetal brachial artery for the collection of fetal blood at 126 DGA.

### Postmortem Examination and Meconium Collection

Pregnant ewes and their fetuses were euthanized with an intravenous dose of pentobarbital sodium (130 mg/kg maternal body weight) administered to the ewe at 134 DGA. Each fetus was immediately removed from the uterus and weighed, and then major fetal organs (kidneys, lungs, heart and brain) were collected and weighed. Several (2–3) placental cotyledons were also collected. Fetal large intestine was removed, cut into proximal (colonic) and distal (rectal segments), and meconium was collected by squeezing the intestine to push out the contents from one end. The samples were then frozen at –80°C, until shipping (on dry ice) to the Hospital for Sick Children, Toronto, Canada for analysis. The subsequent investigations of ethanol-induced pathology in the kidneys [18], lungs [19], heart [17], and placenta and brain [20] were conducted in surgically-instrumented animals that received ethanol and their saline controls (groups 1 and 2 in Table 1), and the findings have been previously reported in the referenced publications. The vascular findings in these fetuses are pending publication. In brief, cerebral vessels from the 1st branch of the middle cerebral artery were isolated and frozen at −60°C. Cerebral vascular tissue was homogenized and RNA extracted using a RNeasy mini kit (Qiagen, Australia). The mRNA was converted to cDNA, and collagen I α1 and tropoelastin mRNA levels quantified using real-time PCR (Realplex Real-Time Multiplexing System, Eppendorf, Germany). Samples were measured in triplicate and a negative control sample which contained no mRNA was included in each PCR run. Fetal mRNA expression levels were normalized against the housekeeping gene 18S rRNA for each sample and analysed using the C_T_ (cycle threshold) method. Values were expressed relative to the mean mRNA expression level in control animals for each gene. This protocol was previously used and reported by our group [19]. Primer sequences, cDNA concentrations, and annealing temperatures that are specific to the vascular tissue, are summarized in the supplement (**Table S1**).

### Meconium Analysis for FAEE

Four FAEE (ethyl palmitate, linoleate, oleate, and stearate) were extracted from the individual meconium samples (two per fetus; one colonic and one rectal) and quantified by headspace solid-phase microextraction gas chromatography-mass spectrometry (HS-SPME GC-MS) using an established methodology [21]. Briefly, FAEE were isolated from 0.5 g meconium via liquid-liquid extraction with heptane:acetone (5∶2, vol/vol), and the extract was concentrated to dryness with a stream of N_2_ gas. The extracted FAEE were quantified using HS-SPME GC-MS with their respective deuterated ethyl esters used as internal standards (prepared as described previously [21]). A gas chromatograph with a mass selective detector GC-MS QP-2010 PLUS equipped with an AOC-5000 autosampler and GCMSsolutions Software (Shimadzu, Columbia, MD) was used for analysis. A 65-µm polydimethylsiloxane/divinylbenzene fiber (Supelco, Bellefonte, PA) and a FactorFour capillary column (Varian, Palo Alto, CA) were used as per established protocol. The calibration curve of each FAEE was linear, and the coefficients of determination (r^2^) ranged from 0.98 to 1.00. The limit of detection (LOD) and limit of quantification (LOQ) of this method for each ester in sheep meconium were determined by construction of a calibration curve in the low concentration range (2.5–40 ng FAEE). The LOD was calculated as three times the ratio between the SD of the linear regression line and the slope of the calibration curve (LOD = 3·SD/*slope*) and the LOQ was determined as ten times the same ratio (LOQ = 10·SD/*slope*). The LOD for the four quantified FAEE ranged from 3.682 to 4.941 ng FAEE/g meconium, and the LOQ was in the range of 12.274 to 16.470 ng FAEE/g meconium. The intra-day coefficient of variation (CV) ranged from 0.5–10.4%, and the inter-day CV ranged from 2–11.3%. The FAEE concentration data were expressed as nanomoles FAEE per gram meconium.

### Data Analysis

All FAEE concentrations that fell below the LOQ could not be reliably quantified, and were assigned a value of zero in all quantitative and statistical analyses. Data analyses were performed with GraphPad Prism 5.0 (GraphPad Prism; San Diego, CA), where more information on the statistical tests used can be found. Since there was no difference in meconium FAEE concentrations between colonic and rectal meconium samples as assessed by the Wilcoxon signed rank test for matched pairs (data shown in **Figure S1** in supplement), the average meconium FAEE concentrations (determined for each fetus by averaging the rectal and colonic FAEE concentrations) were used in all subsequent analyses. Meconium FAEE concentrations were compared between treatment groups using the Kruskal-Wallis test with Dunn’s multiple comparison test, and between all ethanol-exposed (groups 1 and 3 in Table 1 combined) and non-exposed fetuses (groups 2 and 4 in Table 1 combined) with the Mann-Whitney U-test. Concentrations of different FAEE species in meconium of ethanol exposed fetuses were compared using Friedman test with Dunn’s multiple comparison test. A receiver operating characteristic (ROC) curve was constructed, and the area under the ROC curve (AUC) for each FAEE was calculated to assess the ability of meconium FAEE concentration to differentiate between ethanol-exposed and non-exposed fetuses and to identify an optimal positive cut-off value. Sensitivity, specificity, and positive and negative FAEE predictive values were determined for various cut-off values.

The utility of meconium FAEE in identifying fetuses with ethanol-induced pathology was evaluated in surgically-instrumented fetuses exposed to ethanol or saline (groups 1 and 2 in Table 1) by determining the relationship between meconium total FAEE concentration and previously determined specific physiological and/or anatomical endpoints in the organs of these animals. This relationship was assessed as a continuous variable using Spearman correlation analysis, and as a dichotomous variable (positive/negative groupings) by applying the optimal positive cut-off for meconium FAEE concentration and evaluating whether FAEE-positive and FAEE-negative fetuses displayed differences in pathological endpoints using an unpaired Student’s t-test or Mann-Whitney U test. Relationship between meconium FAEE concentration and fetal PEC in instrumented fetuses exposed to ethanol (group 1 in Table 1) was assessed using Spearman correlation analysis. Two groups of data were considered to be statistically different when *p*<0.05.

## Results

### Fetal Growth and Organ Pathology

Ethanol treatment did not significantly alter fetal body weight or absolute organ weights [17]–[20]. However, when adjusted for body weight, heart weight was greater in ethanol exposed fetuses compare to controls [17]. There were no significant differences in the weights of any other organs when adjusted for body weight. Although ethanol-exposed fetuses did not exhibit overt morphological signs of injury, ethanol-induced changes were found in several organs examined as previously described [17]–[20]. These changes will be discussed below in relation to meconium FAEE concentration.

### FAEE in Meconium Samples

In control fetuses (saline and untouched controls), trace levels (above LOD but below LOQ) of mostly one or two measured FAEE species were frequently detectable in at least one of the two collected meconium samples (colonic and/or rectal) from each fetus, but only several fetuses had meconium with quantifiable amounts of any FAEE species (>LOQ), and this was ethyl oleate in all cases (**Tables 2** and **3**). In contrast, meconium of all ethanol-exposed fetuses (instrumented and non-instrumented) contained detectable levels of at least three different FAEE species (**Table 3**). Ethyl oleate, palmitate, and stearate were always detectable (>LOD) and often quantifiable (>LOQ) in at least one of the two samples from each ethanol-exposed fetus (**Table 2**). In contrast, trace amounts of ethyl linoleate were found in meconium samples of both ethanol-exposed and control fetuses, but were quantifiable (>LOQ) in only one ethanol-exposed fetus.

**Table 2:** Number of offspring with undetectable (<LOD), trace (between LOD and LOQ), and quantifiable (>LOQ) FAEE concentrations in meconium (in at least one of two collected samples).

	Undetectable (<LOD)			Trace (LOD-LOQ)			Quantifiable (>LOQ)		
Ethanol-exposed groups^a^	1 (n = 9)	3 (n = 6)	All (n = 15)	1 (n = 9)	3 (n = 6)	All (n = 15)	1 (n = 9)	3 (n = 6)	All (n = 15)
Ethyl Palmitate (E16∶0)	0	0	0 (0%)	6	1	7 (46.7%)	3	5	8 (53.3%)
Ethyl Linoleate (E18∶2)	1	3	4 (26.7%)	7	3	10 (66.7%)	1	0	1(6.7%)
Ethyl Oleate (E18∶1)	0	0	0 (0%)	1	1	2 (13.3%)	8	5	13 (86.7%)
Ethyl Stearate (E18∶0)	0	0	0 (0%)	3	0	3 (20.0%)	6	6	12 (80.0%)
**Control groups** b	**2 (n = 9)**	**4 (n = 6)**	**All (n = 15)**	**2 (n = 9)**	**4 (n = 6)**	**All (n = 15)**	**2 (n = 9)**	**4 (n = 6)**	**All (n = 15)**
Ethyl Palmitate (E16∶0)	9	6	15 (100%)	0	0	0 (0%)	0	0	0 (0%)
Ethyl Linoleate (E18∶2)	4	2	6 (40%)	5	4	9 (60%)	0	0	0 (0%)
Ethyl Oleate (E18∶1)	1	4	5 (33.3%)	6	1	7 (46.7%)	2	1	3 (20.0%)
Ethyl Stearate (E18∶0)	8	4	12 (80%)	1	2	3 (20%)	0	0	0 (0%)

a Surgically-instrumented and non-surgically-instrumented fetuses of surgically-instrumented pregnant ewes that received ethanol treatment (groups 1 & 3, respectively).

b Surgically-instrumented fetuses of surgically-instrumented ewes that received saline treatment and non-surgically-instrumented fetuses of untouched ewes (groups 2 & 4, respectively).

**Table 3:** Number of offspring with detectable (>LOD) and quantifiable (>LOQ) number of different FAEE species in meconium (in at least one of two collected samples).

	Ethanol Groups^a^						Control Groups^b^					
	Detectable (>LOD)			Quantifiable (>LOQ)			Detectable (>LOD)			Quantifiable (>LOQ)		
# of FAEE	1 (n = 9)	3 (n = 6)	All (n = 15)	1 (n = 9)	3 (n = 6)	All (n = 15)	2 (n = 9)	4 (n = 6)	All (n = 15)	2 (n = 9)	4 (n = 6)	All (n = 15)
**0**	0	0	0 (0%)	0	0	0 (0%)	0	1	1 (6.7%)	7	5	12 (80.0%)
**1**	0	0	0 (0%)	4	1	5 (33.3%)	5	3	8 (53.3%)	2	1	3 (20.0%)
**2**	0	0	0 (0%)	2	0	2 (13.3%)	3	1	4 (26.7%)	0	0	0 (0%)
**3**	1	3	4 (26.7%)	2	5	7 (46.7%)	1	1	2 (13.3%)	0	0	0 (0%)
**4**	8	3	11 (73.3%)	1	0	1 (6.7%)	0	0	0 (0%)	0	0	0 (0%)

a Surgically-instrumented and non-surgically-instrumented fetuses of surgically-instrumented pregnant ewes that received ethanol treatment (groups 1 & 3, respectively).

b Surgically-instrumented fetuses of surgically-instrumented ewes that received saline treatment and non-surgically-instrumented fetuses of untouched ewes (groups 2 & 4, respectively).

**Figure 1:**
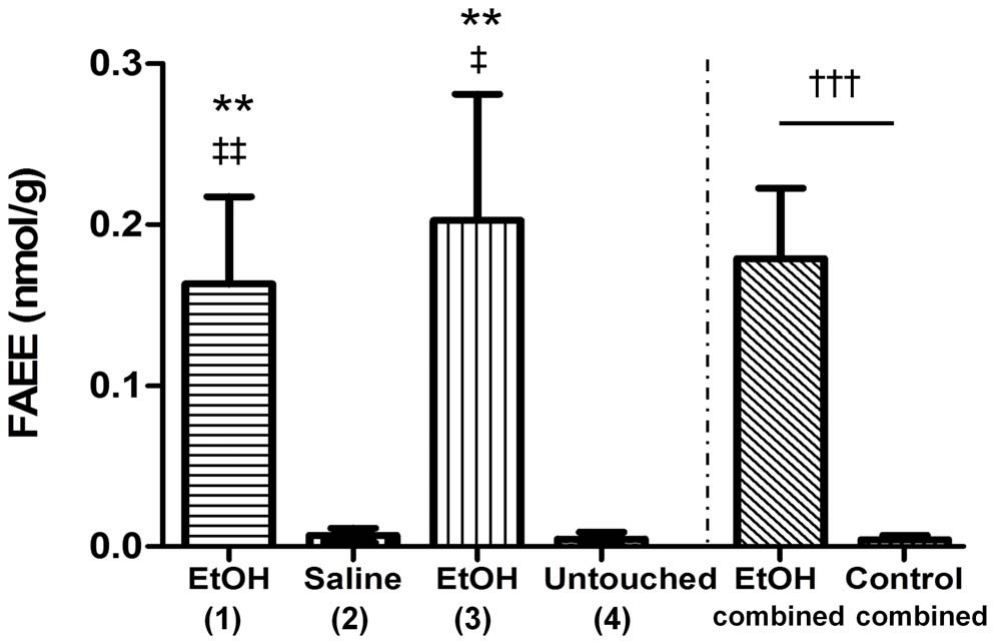
Effect of daily ethanol exposure in late gestation on FAEE concentration in fetal meconium. Bars depict FAEE concentration (sum of four) in meconium (averaged rectal and colonic concentrations for each fetus) of ethanol-exposed (groups 1 and 3) and non-exposed fetuses (groups 2 and 4). Data are presented as mean +/− SEM, *n* = 6–9 per treatment group, *n* = 15 for combined ethanol and control groups. Kruskal-Wallis test with Dunn’s multiple comparison test was used to compare FAEE concentrations between the four different treatment groups. No statistically significant differences in FAEE concentrations were found between untouched and saline controls (groups 2 and 4) or between surgically instrumented and non-instrumented fetuses exposed to ethanol (groups 1 and 3) (*p*>0.05). FAEE concentrations were significantly higher in the two ethanol-exposed groups (1 and 3) compare to the two control groups (2 and 4) (**p<0.01 compare to saline, *‡p*<0.05 and ‡‡ *p*<0.01 compare to untouched control). Mann-Whitney U test was used to compare the average concentration between all ethanol-exposed (groups 1 and 3 combined) and non-exposed fetuses (groups 2 and 4 combined). FAEE concentrations were significantly higher in ethanol-exposed fetuses compare to unexposed controls (†††*p*<0.0001).

**Figure 2:**
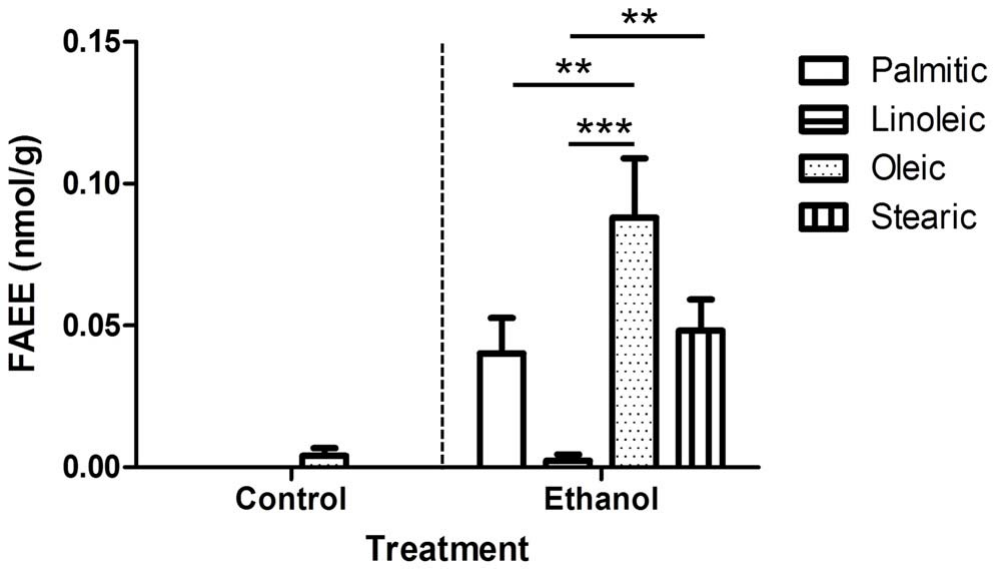
Concentration of different FAEE species in meconium from ethanol-exposed and control fetuses. Data are presented as mean +/− SEM, *n* = 15 per treatment group. FAEE concentrations were compared using Friedman test with Dunn’s multiple comparison test (**p<0.01,***p<0.001).

As mentioned above, since there was no statistically significant difference between FAEE concentrations in colonic and rectal meconium samples (**Figure S1** in supplement), average meconium concentrations for each fetus were used in comparisons of meconium FAEE content between treatment groups. Meconium FAEE concentrations were significantly higher in the two ethanol-exposed groups (1 and 3) compare to the two control (non-exposed) groups (2 and 4), and there were no statistically significant differences in FAEE concentrations between untouched and saline controls (groups 2 and 4) or between surgically instrumented and non-instrumented fetuses exposed to ethanol (groups 1 and 3) (**Figure 1**). Since instrumentation did not appear to affect meconium FAEE concentration (and was not expected to, biologically), the two ethanol groups and the two control groups were combined and compared based on treatment received (ethanol vs. no ethanol), which again showed significantly higher meconium FAEE concentrations in the ethanol-exposed fetuses compare to the non-exposed controls (*p*<0.0001) (**Figure 1**). Mean meconium FAEE concentration (average of rectal and colonic meconium FAEE concentrations of each fetus) in ethanol-exposed fetuses was 0.179 nmol/g (median: 0.108, range: 0.020–0.561) compared with 0.004 nmol/g (median: 0, range: 0–0.034) in control fetuses. In meconium of ethanol-exposed fetuses, ethyl oleate was generally found at higher concentrations than other measured FAEE species, followed by stearate and palmitate (**Figure 2**).

### ROC Analysis: Sensitivity and Specificity

**Figure 3:**
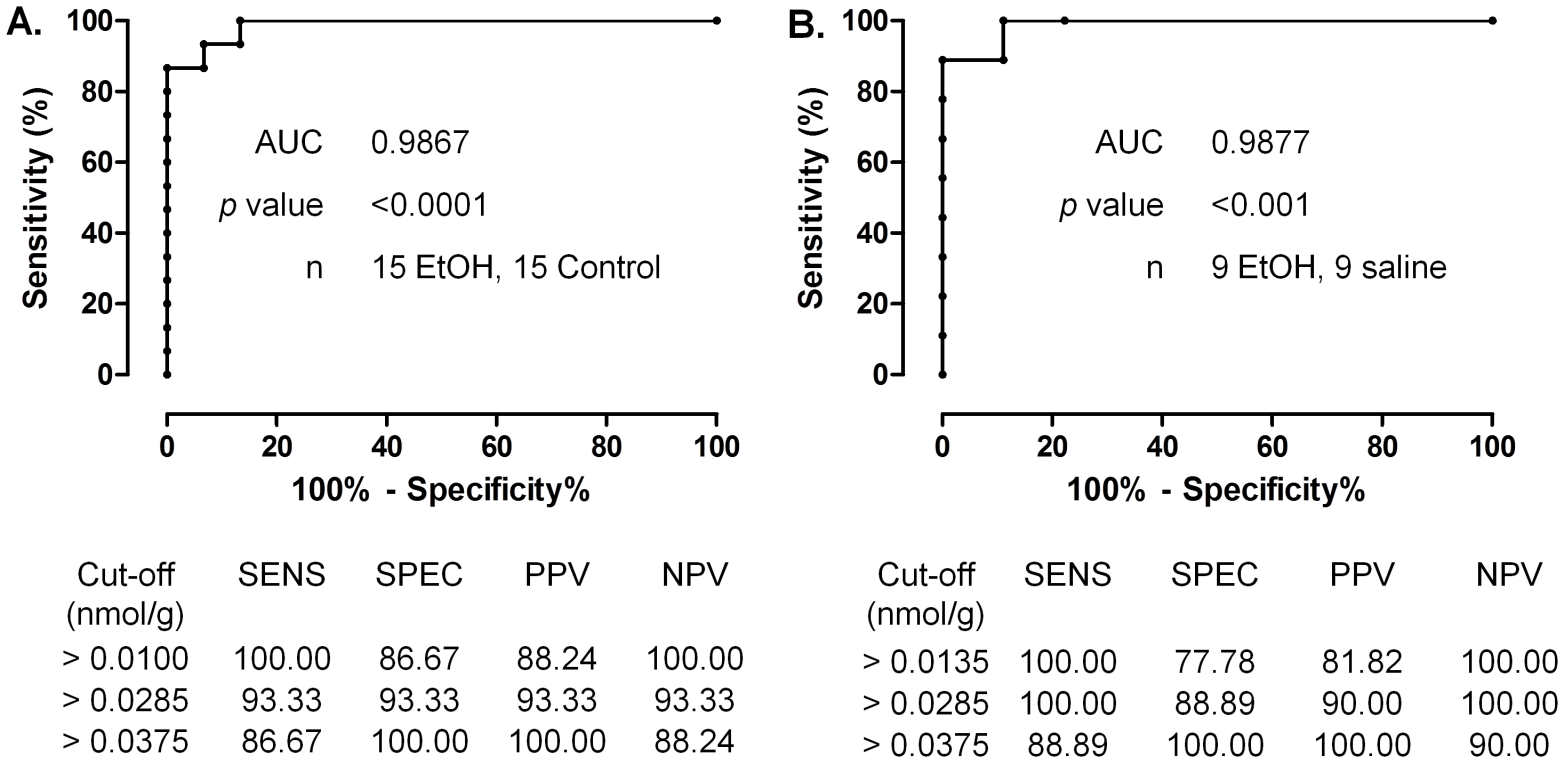
Receiver operating characteristic (ROC) curve depicting the sensitivity and specificity of total meconium FAEE concentration (sum of four) for the identification of late-gestation fetal ethanol exposure utilized in this study. (**A**) Results of ROC curve analysis using meconium FAEE values from all ethanol exposed (groups 1 and 3) and non-exposed fetuses (groups 2 and 4), and (**B**) using meconium FAEE data from only surgically instrumented fetuses exposed to ethanol or saline (groups 1 and 2). Sensitivity, specificity, positive and negative predictive values (PPV and NPV, respectively) for the best positive cut-off values are displayed below the curves.

The ability of meconium FAEE concentration to identify the ethanol exposure regimen used in this study was determined by ROC analysis using data from all ethanol-exposed and control (saline and untouched) fetuses and using data from instrumented fetuses only (groups 1 and 2 in Table 1).The AUCs of the ROC curves for each FAEE are presented in **Table 4**. The sum of four FAEE had the highest AUC value of >0.98 in both cases, which was significantly different from 0.5, indicating excellent ability of the test to discriminate between ethanol-exposed and non-exposed offspring. No single FAEE species and no other combination of FAEE had a higher AUC value, and thus, the sum of the four FAEE was used in all subsequent analyses. The ROC curves for sum of four FAEEs along with the cut-off values associated with the highest sensitivity and specificity for detecting fetal ethanol exposure are depicted in **Figure 3**. Using data from all ethanol exposed animals or limiting it to instrumented fetuses receiving ethanol or saline (groups 1 and 2, respectively) yielded virtually identical ROC curves and results (**Figure 3**). The positive cut-off value of 0.0285 nmol FAEE/g meconium had 93% sensitivity and specificity for detecting fetal ethanol exposure in the studied animals. Since this positive cut-off value offered the optimum balance between sensitivity and specificity, it was chosen to be used in subsequent analyses assessing the relationship between meconium FAEE and fetal organ abnormalities.

**Table 4:** ROC curve analysis of meconium FAEE.

	Data from all ethanol exposed^a^ and non-exposed^b^ fetuses			Data from instrumented fetuses receiving ethanol or saline (groups 1 and 2)		
	AUC	95% CI	*p* value	AUC	95% CI	*p* value
**Ethyl Palmitate (E16∶0)**	0.8333	0.6768 to 0.9899	<0.01	0.7778	0.5499 to 1.006	<0.05
**Ethyl Oleate (E18∶1)**	0.9356	0.8429 to 1.0280	<0.0001	0.9259	0.8046 to 1.047	<0.01
**Ethyl Stearate (E18∶0)**	0.9333	0.8285 to 1.0380	<0.0001	0.8889	0.7166 to 1.061	<0.01
**Ethyl Linoleate (E18∶2)**	0.5333	0.3237 to 0.7429	0.7557	0.5556	0.2832 to 0.8280	0.691
**TOTAL**	0.9867	0.9575 to 1.0160	<0.0001	0.9877	0.9489 to 1.026	<0.001

a Surgically-instrumented and non-surgically-instrumented fetuses of surgically-instrumented pregnant ewes that received ethanol treatment (groups 1 & 3, respectively).

b Surgically-instrumented fetuses of surgically-instrumented ewes that received saline treatment and non-surgically-instrumented fetuses of untouched ewes (groups 2 & 4, respectively).

### Relationship between Meconium FAEE and Fetal Organ Abnormalities

#### Kidney

In the fetal kidneys, ethanol exposure resulted in an 11% decrease in nephron endowment with no apparent change in overall kidney growth [18]. Accordingly, when the studied animals (instrumented ethanol-exposed fetuses and saline controls) were classified using their meconium FAEE concentration, those testing above the chosen positive cut-off for meconium FAEE (>0.0285 nmol FAEE/g meconium), as a group, had a lower nephron number compared with fetuses testing below the positive cut-off value for FAEE. Furthermore, in these animals as a group (ethanol-exposed and saline controls combined), there was a negative correlation between meconium FAEE concentration and nephron number in fetal kidneys (**Table 5**).

**Table 5:** Relationship between meconium FAEE concentration and ethanol-induced changes in fetal organs (endpoint).

Organ endpoint	Meconium group assignment			Correlation with meconium FAEE content†	
	FAEE-negative (<0.0285 nmol/g)	FAEE-positive (>0.0285 nmol/g)	*p*-value*	r_s_	*p*-value
**Kidney** a					
Nephron number	427706±8012	382251±6731	p<0.01	−0.7289	p<0.05
**Lung** b					
Collagen deposition (% collagen/total area)	21.42±0.31	25.84±1.41	p<0.05‡	0.5783	p<0.05
COLIα1 mRNA level	1.05±0.30	1.63±0.23	p = 0.094	0.4437	p = 0.098
SP-A mRNA level	0.87±0.26	0.56±0.21	p = 0.285	−0.1734	p = 0.537
SP-B mRNA level	1.00±0.23	0.52±0.09	p = 0.051	−0.5662	p<0.05
IL-1β mRNA level	0.72±0.43	0.47±0.36	p = 0.286	−0.4297	p = 0.110
IL-8 mRNA level	0.67±0.39	0.55±0.40	p = 0.344	−0.2305	p = 0.408
**Heart** c					
Heart weight/body weight (g/kg)	6.8±0.4	8.7±0.7	p = 0.073	0.5711	p<0.05
LV+S wall volume/body weight (mm^3^/kg)	3.29±0.17	3.69±0.11	p = 0.069	0.5265	p = 0.064
% mononucleated cardiomyocytes within LV+S	35.8±1.8	25.3±1.9	p<0.01	−0.6491	p<0.05
% binucleated cardiomyocytes within LV+S	64.2±1.8	74.7±1.9	p<0.01	0.6491	p<0.05
**Cerebral vessels**					
Tropoelastin mRNA level	1.08±0.09	1.56±0.10	p<0.01	0.8211	p<0.01
COLIα1 mRNA level	1.07±0.14	1.47±0.19	p = 0.126	0.7176	p<0.05
**Placenta** d					
TNF-α mRNA level	1.17±0.19	1.80±0.32	p = 0.113	0.5926	p<0.05

*Note:* Pathology analyses were conducted in instrumented animals receiving ethanol and their appropriate instrumented controls that received saline (groups 1 and 2 listed in Table 1). Organ pathology findings based on treatment group assignment (ethanol vs. control) have been previously reported in their respective publications, which are cited for each organ of interest. Herein, the animals are grouped according to their meconium FAEE concentrations, rather than treatment received. All animal pathology data is displayed as mean ± SEM, with an *n* value of 4–9 fetuses per group depending on the outcome in question. The mRNA data are presented as fold change relative to control. Lung mRNA data were log-transformed prior to statistical analysis. Spearman correlation analysis between meconium FAEE concentration and anatomical/physiological endpoints in individual animals was conducted using data from ethanol-exposed fetuses and saline controls, with an *n* of 9–15 fetuses depending on the endpoint in question. Abbreviations: Collagen I α1 (COLIα1), surfactant protein (SP), interleukin (IL), left ventricle plus septum (LV+S), tumour necrosis factor alpha (TNF-α).

a For detailed methodology and results based on treatment received, see [18].

b For detailed methodology and results based on treatment received, see [19].

c For detailed methodology and results based on treatment received, see [17].

d For detailed methodology and results based on treatment received, see [20].

* Unpaired Student’s t-test unless otherwise specified.

‡ Mann-Whitney U test.

† Spearman correlation.

#### Lung

Ethanol exposure led to a 75% increase in collagen I α1 mRNA level and significantly increased collagen deposition in fetal lungs [19]. Similarly, we found that when the studied animals (instrumented ethanol-exposed fetuses and saline controls) were divided into meconium FAEE-positive and FAEE-negative groups using the positive cut-off of 0.0285 nmol/g, fetuses that tested above this value had significantly more collagen deposition (**Table 5**). When meconium FAEE concentration was considered as a continuous variable in these animals (ethanol-exposed and saline controls combined), there was a positive correlation between meconium FAEE concentration and increased collagen deposition in fetal lungs. Significantly decreased mRNA levels of surfactant protein (SP)-A and SP-B, and proinflammatory cytokines interleukin (IL)-1β and IL-8 in the lungs of ethanol-exposed fetuses were also reported [19]. Accordingly, a significant negative correlation was observed between meconium FAEE concentration and SP-B mRNA expression in these animals as a group. This was not the case for SP-A or interleukin mRNA expression.

#### Heart

In the fetal heart, ethanol exposure resulted in an increase in relative heart weight and left ventricle wall volume [17]. When FAEE concentration was considered as continuous variable in these animals (instrumented ethanol-exposed fetuses and saline controls combined), there was a significant positive correlation between meconium FAEE concentration and relative heart weight (**Table 5**). However, when the studied animals were classified according to their meconium FAEE concentration, the difference in relative heart weight and left ventricle wall volume in FAEE-positive fetuses compared with FAEE-negative fetuses did not reach statistical significance (*p* = 0.073 and 0.069, respectively) (**Table 5**). Ethanol exposure was also shown to advance cardiomyocyte maturation in the left ventricle of these fetal sheep [17], and, accordingly, there was a significantly higher proportion of binucleated cardiomyocytes and lower proportion of mononucleated cardiomyocytes within the left ventricle and septum in FAEE-positive compared with FAEE-negative fetuses. Furthermore, there was a significant positive correlation of meconium FAEE concentration with binucleated cardiomyocytes and a negative correlation with mononucleated cardiomyocytes within the left ventricle and septum in the studied animals as a group (**Table 5**).

#### Brain

In the fetal brain, ethanol exposure increased mRNA levels of tropoelastin and collagen I α1 in small cerebral vessels (unpublished observations), and three out of eight ethanol-exposed fetuses examined for brain pathology exhibited small subarachnoid hemorrhages in the cerebrum and/or cerebellar parenchyma associated with focal cortical neuronal cell death and gliosis [20]. Accordingly, FAEE-positive fetuses (>0.0285 nmol/g) had significantly elevated level of tropoelastin mRNA compared with FAEE-negative fetuses, although the collagen I α1 mRNA levels in FAEE-positive fetuses were not significantly different from the FAEE-negative fetuses (**Table 5**). Furthermore, in the studied animals as a group (exposed and control fetuses combined), meconium FAEE concentration correlated positively with both tropoelastin and collagen I α1 mRNA levels in cerebral vessels (**Table 5**). The three fetuses in the ethanol group with histologically identified hemorrhages in the cerebrum and/or cerebellum had meconium FAEE concentrations ranging from 0.041 to 0.094 nmol/g and, therefore, were identifiable as FAEE-positive based on the established cut-off.

#### Placenta

In the placenta, ethanol exposure elevated mRNA level of tumor necrosis factor (TNF)-α, suggesting a persistent inflammatory response in this tissue [20]. Although there was a positive, statistically significant correlation between meconium FAEE concentration and mRNA expression of TNF-α in the placentas of ethanol-exposed fetuses and controls as a group, when animals were divided into meconium FAEE-positive and FAEE-negative groups using the 0.0285 nmol FAEE/g meconium cut-off value, there was no statistical difference between the TNF-α mRNA expression values for the two groups (**Table 5**).

### Relationship between Meconium FAEE and Fetal PEC

In instrumented fetuses exposed to ethanol (group 1 in Table 1), there was no statistically significant correlation between the total meconium concentration of FAEE and maximal PEC achieved in individual fetuses at the end of the 1-hr maternal ethanol infusion, or with AUC of the ethanol-concentration-time-curve in individual fetuses measured on 131–134 DGA (Spearman r = 0.028, *p* = 1.000).

## Discussion

The present study evaluated the usefulness of measuring meconium FAEE following ethanol exposure of fetal sheep in late gestation that does not cause abnormal physical development, but produces subtle, important pathology in major organs. Of specific interest was the relationship between this biomarker of fetal ethanol exposure and organ pathology (effect) as an assessment of its ability to identify ethanol-affected newborns. The late gestation ethanol regimen is relevant because it has been shown that women may be more likely to drink in the third trimester than throughout pregnancy in response to the commonly-held misconception that alcohol consumption is less harmful to the fetus later in pregnancy [22], [23]. However, the third trimester is a period of structural and functional maturation of many organs, including the brain, which undergoes a growth spurt peaking at parturition in humans, and at approximately 133 DGA in sheep [24]. The observed alterations in fetal organs induced by ethanol exposure during this gestational time period may lead to sub-optimal organ function and susceptibility to dysfunction, especially if challenged by other risk factors commonly seen in ethanol-exposed neonates (e.g., prematurity, low birth weight, poor nutritional status). For example, decreased nephron endowment and altered maturation of the heart may have implications for renal and cardiovascular function post-natally; increased collagen deposition and decreased expression of surfactants and interleukins in the lungs may decrease distensibility and predispose to infection; and increased expression of tropoelastin and collagen in cerebral vasculature may increase risk of hemorrhage. Furthermore, the effect that these ethanol-induced system-wide changes can have on the brain may be of developmental and functional significance.

The fetal sheep is a suitable model for studying ethanol teratogenicity and validation of biomarkers because of similarities in organ development and comparable ethanol disposition with the human fetus. In both sheep and humans, ethanol readily distributes across the placenta and the pharmacokinetics of maternal-fetal distribution and elimination are similar [25], [26], with ethanol clearance predominantly regulated by its oxidative metabolism in the maternal liver [27]. Furthermore, the ability of the ovine fetus to synthesize FAEE is likely comparable to the human fetus since a previous study in fetal sheep demonstrated that high level/binge exposure to ethanol in late gestation resulted in FAEE concentration of comparable magnitude to that observed in heavily ethanol-exposed human neonates [28].

The present study demonstrated that, in sheep, fetal meconium FAEE concentration can serve as a biomarker of chronic ethanol exposure in the third trimester that results in peak PEC frequently observed in non-pregnant women drinking socially [13]. Furthermore, a positive cut-off could be established to identify ethanol-exposed fetuses with high sensitivity and specificity, and thereby detect those with ethanol-induced pathological abnormalities. As expected, the total concentration of FAEE (sum of 4) found in meconium of ethanol-exposed sheep was relatively low; ∼10 to 100-fold lower than the 2 nmol/g positive cut-off proposed for identifying heavy ethanol exposure in human newborns [29]. When the studied animals (instrumented ethanol-exposed fetuses and saline controls) were divided into FAEE-positive and FAEE-negative groups, these differed in numerous pathological endpoints in fetal organs, including nephron endowment, lung collagen deposition, cardiomyocyte maturation, and tropoelastin gene expression in cerebral vasculature. Furthermore, meconium FAEE concentration correlated with many of the examined pathological endpoints when assessed as a continuous variable in the studied animals as a group, and we cannot exclude that the small number of animals studied for organ toxicity may have precluded statistical significance in some analyses (potential type II error). These results suggest that meconium FAEE may be useful in identifying newborns at risk for ethanol-induced organ and system dysfunction, who may not exhibit overt signs of organ malformations or injury.

In the present study, we evaluated the four FAEE species the sum of which has been shown to have the highest sensitivity and specificity for detecting prenatal ethanol exposure in humans [29]. We found that using a combination of the four FAEE species was advantageous over the use of any single FAEE in identifying ethanol-exposed sheep fetuses, and possessed the highest area under the curve in ROC analysis. Of the different FAEE species, ethyl oleate and stearate possessed comparably high specificities and sensitivities in identifying ethanol exposure, but the combination of FAEE was still superior to any single FAEE alone. These results are in agreement with several studies that have reported combined FAEE concentration as a better biomarker of prenatal ethanol exposure [29]–[31]; but are in contrast to other studies that suggest the use of one particular FAEE (e.g. ethyl oleate or linoleate) [12], [13], [28], [32]. Interestingly, we found mostly trace amounts of ethyl linoleate in meconium of ethanol-exposed sheep and some controls, and this ester had the lowest area under the curve in ROC analysis of the four measured FAEE. This contrasts with some previous animal and human studies that have found this FAEE to be a prominent in meconium of ethanol-exposed newborns [12], [28]. This may be due to species/breed and diet differences, as well as different ethanol exposure patterns, which can all potentially affect formation of specific FAEE species. Moreover, ethyl linoleate may be less stable in meconium than other FAEE due to its polyunsaturated chemical structure, which could make it more susceptible to degradation during shipping and storage, similarly to ethyl arachidonate [33].

One previous study investigated meconium FAEE concentration in fetal sheep prenatally exposed to ethanol [28]. Pregnant ewes were infused with ethanol in late gestation, but higher doses of ethanol were used (1.25–2.00 g/kg maternal body weight; unreported PEC). As in the present study, concentrations of several FAEE species were significantly higher in ethanol-exposed animals compared with controls, but the concentrations were several-fold higher than in the present study, as would be expected due to reporting of FAEE concentrations per dry meconium weight rather than wet weight, and the higher ethanol dosage used. In contrast to our study, however, significant FAEE concentration in some control sheep was found, which may be due to animal breed (Suffolk *vs*. Merino x Border Leicester) and/or diet differences leading to variation in endogenous ethanol levels and thus, variable baseline FAEE concentration. Differences also could be due to the use of different FAEE analytical procedures; the less selective GC-flame ionization detector method used by Littner and colleagues may be more prone to “false positives” compared with the GC-MS procedure used in the present study [34], [35].

Maternal and fetal instrumentation, which was necessary for ethanol/saline administration and PEC measurements in this study, was not a likely confounding factor with regard to meconium FAEE concentrations. Since groups 1 and 3 were exposed to the same ethanol dosing regimen, we did not expect and did not find differences in meconium FAEE concentrations between the two groups. Similarly, we did not find differences in baseline FAEE concentrations between groups 2 and 4 (instrumented animals receiving saline and untouched controls), none of which received ethanol– the primary determinant of FAEE levels in tissues. In contrast, the difference in meconium FAEE concentrations between groups that received ethanol and those that did not, was significant. Additionally, we confirmed that inclusion of non-instrumented fetuses did not affect the results of ROC curve sensitivity/specificity analysis and yielded essentially the same optimal cut- off values, thus further confirming that instrumentation was not a likely confounding factor with respect to meconium FAEE concentrations.

Using meconium FAEE as a biomarker has numerous advantages. Meconium is a cumulative matrix that provides a record of xenobiotic exposure over a relatively long time; it can be collected non-invasively; and sensitive methods have been developed to measure FAEE in meconium [13], [21], [36]. Additionally, human studies exist that have reported association between elevated meconium FAEE concentration and lower APGAR scores [37], growth restriction and decreased executive functioning [38], and poor mental and psychomotor development [39]. Thus, the results of this and previous studies are highly encouraging that meconium FAEE may be well-suited for identifying newborns at risk for disabilities due to prenatal ethanol exposure. Another advantage is that meconium FAEE are a product of fetal ethanol burden because maternally produced FAEE do not cross the placenta [40]. Of interest, we found no correlations between meconium FAEE concentration in individual fetuses and peak PEC or AUC of the fetal PEC-time curve. Thus, the observed variability in meconium FAEE among fetuses in the ethanol group may reflect individual differences in ethanol metabolism, FAEE baseline concentration, content of fatty acids, abundance and kinetics of enzymes with FAEE-synthetic activity, and the rate of FAEE breakdown.

There are some challenges to using meconium FAEE as a biomarker of fetal ethanol exposure. Since meconium begins to form in the human fetal intestine in the second trimester (15–20 weeks gestation) and accumulates mostly in the latter part of pregnancy [34], prenatal alcohol exposure earlier in pregnancy may not be detectable if the mother later abstained from drinking. The finding that there was no significant difference in total FAEE concentration between colonic and rectal meconium may indicate that, similarly to humans, a large proportion of meconium is formed and deposited towards the end of pregnancy (when ethanol exposure occurred in the current study). There are also some potential limitations to using sheep as an animal model. Firstly, although in humans it is known that FAEE do not cross the placenta and are thus indicative of fetal ethanol load, such studies have not been conducted in sheep placentas. Secondly, humans and sheep have different diets which could affect the fatty acid composition in fetal blood and thus the FAEE profiles. Thirdly, sheep are ruminant animals capable of producing small amounts of ethanol from glycerol fermentation [41], which could explain the presence of FAEE in controls. However, how much this differs from endogenous ethanol production in the human gut is unknown. Thus, although we were able to establish a highly sensitive and specific FAEE cut-off that could detect relatively moderate fetal ethanol exposure in this animal study, large population-based studies are required to determine the sensitivity and specificity of meconium FAEE for detecting various patterns of alcohol use in human pregnancy.

We conclude that meconium FAEE concentration can serve as a reliable biomarker of fetal ethanol exposure in late ovine gestation, and can be used as an indicator of abnormalities in fetal organ development. These findings are encouraging as they suggest that meconium FAEE may identify newborns at risk of multi-organ dysfunction, who may not be displaying overt physical signs of ethanol teratogenicity. Whether it will be possible to set a lower positive cut-off value in the human than the current 2 nmol FAEE/g meconium to identify individuals at risk for more subtle ethanol-related disabilities remains to be determined through human studies.

## Supporting Information

Figure S1 **Effect of daily ethanol exposure in late gestation on FAEE concentration in fetal rectal and colonic meconium.** Bars depict FAEE concentration (sum of four) in meconium collected from the rectal and colonic portions of the large intestine, as well as their average, in ethanol-exposed (groups 1 and 3) and control (groups 2 and 4) fetuses. Data are presented as mean +/− SEM, *n* = 15 per treatment group. There was no statistically significant difference in meconium FAEE concentration between colonic and rectal meconium samples as assessed by the Wilcoxon signed rank test for matched pairs (*p*>0.05). Kruskal-Wallis test with Dunn’s multiple comparison test, which was used to compare FAEE concentrations in colonic and rectal samples between ethanol exposed and non-exposed fetuses, showed significantly higher FAEE concentrations in both colonic and rectal meconium of ethanol exposed fetuses compare to controls (**p<0.01; ***p<0.001). Mann-Whitney U test was used to compare the average meconium FAEE concentration (colonic and rectal concentrations averaged for each fetus) between the two treatment groups and yielded the same result (^†††^ *p*<0.001).
(TIF)

Table S1 **Forward and reverse primer sequences (5′-3′) used for qPCR to amplify** ***Collagen Iα1*** **and** ***tropoelastin.***
(DOC)
