# Supplementary figures and images for: Meconium Fatty Acid Ethyl Esters as Biomarkers of Late Gestational Ethanol Exposure and Indicator of Ethanol-Induced Multi-Organ Injury in Fetal Sheep
Source: PLoS One. 2013 Mar 22;8(3):e59168. doi: 10.1371/journal.pone.0059168 (PMC3606447; doi:10.1371/journal.pone.0059168)

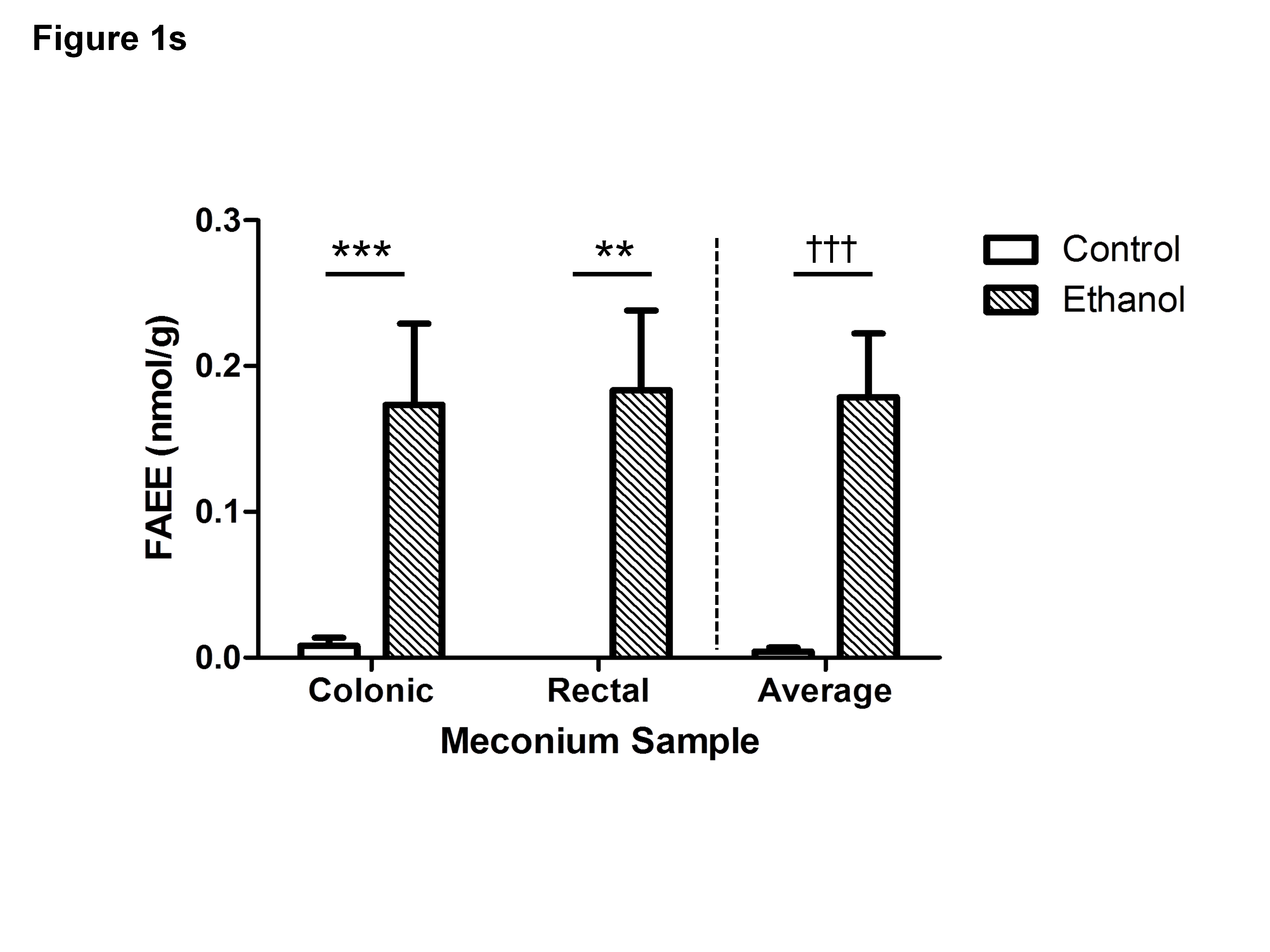

Supplement: Figure S1 — Effect of daily ethanol exposure in late gestation on FAEE concentration in fetal rectal and colonic meconium. Bars depict FAEE concentration (sum of four) in meconium collected from the rectal and colonic portions of the large intestine, as well as their average, in ethanol-exposed (groups 1 and 3) and control (groups 2 and 4) fetuses. Data are presented as mean +/− SEM, n = 15 per treatment group. There was no statistically significant difference in meconium FAEE concentration between colonic and rectal meconium samples as assessed by the Wilcoxon signed rank test for matched pairs (p>0.05). Kruskal-Wallis test with Dunn’s multiple comparison test, which was used to compare FAEE concentrations in colonic and rectal samples between ethanol exposed and non-exposed fetuses, showed significantly higher FAEE concentrations in both colonic and rectal meconium of ethanol exposed fetuses compare to controls (**p<0.01; ***p<0.001). Mann-Whitney U test was used to compare the average meconium FAEE concentration (colonic and rectal concentrations averaged for each fetus) between the two treatment groups and yielded the same result (††† p<0.001). (TIF) [file pone.0059168.s001.tif]
