# Supplementary material for: Meconium Fatty Acid Ethyl Esters as Biomarkers of Late Gestational Ethanol Exposure and Indicator of Ethanol-Induced Multi-Organ Injury in Fetal Sheep
Source: PLoS One. 2013 Mar 22;8(3):e59168. doi: 10.1371/journal.pone.0059168 (PMC3606447; doi:10.1371/journal.pone.0059168)
Supplement: Table S1 — Forward and reverse primer sequences (5′-3′) used for qPCR to amplify Collagen Iα1 and tropoelastin. (DOC) [file pone.0059168.s002.doc]

**Table S1. Forward and reverse primer sequences (5’-3’) used for qPCR to amplify *Collagen Iα1* and *tropoelastin.***

| **Gene** |  | **Primer sequence** | **Primer concentration** | **cDNA concentration** | **Annealing temperature** |
| --- | --- | --- | --- | --- | --- |
| *Collagen I α1* | Forward | aagacatcccaccagtcacc | 10μM | 500ng | 60°C |
|  | Reverse | cagatcacgtcatcgcaca |  |  |  |
| *Tropoelastin* | Forward | atctctcagtcaggcaccag | 10μM | 1000ng | 58°C |
|  | Reverse | gtttgttgggaaagaaagca |  |  |  |
